# Organisational culture and post-merger integration in an academic health centre: a mixed-methods study

**DOI:** 10.1186/s12913-014-0673-3

**Published:** 2015-01-22

**Authors:** Pavel V Ovseiko, Karen Melham, Jan Fowler, Alastair M Buchan

**Affiliations:** Medical Sciences Division, University of Oxford, John Radcliffe Hospital, OX3 9DU Oxford, UK; Centre for Health Law and Emerging Technologies (HeLEX), Department of Population Health, University of Oxford, Oxford, UK; Research Services, University of Oxford, Oxford, UK; NHS England, Thames Valley, UK; Oxford University Hospitals NHS Trust, Oxford, UK

**Keywords:** Organisational culture, Competing Values Framework (CVF), Post-merger integration, University Hospital, Academic Health Centre (AHC), Academic-Clinical Collaboration, Strategic partnership, Research and innovation, Teaching, Patient care

## Abstract

**Background:**

Around the world, the last two decades have been characterised by an increase in the numbers of mergers between healthcare providers, including some of the most prestigious university hospitals and academic health centres. However, many mergers fail to bring the anticipated benefits, and successful post-merger integration in university hospitals and academic health centres is even harder to achieve. An increasing body of literature suggests that organisational culture affects the success of post-merger integration and academic-clinical collaboration.

**Methods:**

This paper reports findings from a mixed-methods single-site study to examine 1) the perceptions of organisational culture in academic and clinical enterprises at one National Health Service (NHS) trust, and 2) the major cultural issues for its post-merger integration with another NHS trust and strategic partnership with a university. From the entire population of 72 clinician-scientists at one of the legacy NHS trusts, 38 (53%) completed a quantitative Competing Values Framework survey and 24 (33%) also provided qualitative responses. The survey was followed up by semi-structured interviews with six clinician-scientists and a group discussion including five senior managers.

**Results:**

The cultures of two legacy NHS trusts differed and were primarily distinct from the culture of the academic enterprise. Major cultural issues were related to the relative size, influence, and history of the legacy NHS trusts, and the implications of these for respective identities, clinical services, and finances. Strategic partnership with a university served as an important ameliorating consideration in reaching trust merger. However, some aspects of university entrepreneurial culture are difficult to reconcile with the NHS service delivery model and may create tension.

**Conclusions:**

There are challenges in preserving a more desirable culture at one of the legacy NHS trusts, enhancing cultures in both legacy NHS trusts during their post-merger integration, and in aligning academic and clinical cultures following strategic partnership with a university. The seeds of success may be found in current best practice, good will, and a near identical ideal of the future preferred culture. Strong, fair leadership will be required both nationally and locally for the success of mergers and post-merger integration in university hospitals and academic health centres.

**Electronic supplementary material:**

The online version of this article (doi:10.1186/s12913-014-0673-3) contains supplementary material, which is available to authorized users.

## Background

Around the world, the last two decades have been characterised by an increase in the numbers of mergers between healthcare providers, including some of the most prestigious university hospitals and academic health centres (AHCs) [1-4]. An AHC consists of an academic enterprise represented by a medical school and other health profession schools or programmes, and an owned or affiliated clinical enterprise represented by one or more hospitals or health systems. Most commonly, an AHC is not a single institution, but “a constellation of functions and organizations committed to improving the health of patients and populations through the integration of their roles in research, education, and patient care” [5]. Because of their unique tripartite roles, AHCs have been at the forefront of innovation and high-quality care in North America and have spread internationally [6].

In England, the government has also attempted to improve efficiency, innovation, and the quality of care in the NHS, *inter alia*, through the integration of health care providers and the promotion of NHS/University partnerships [7]. Recent government policies to convert all hospitals into more financially sustainable Foundation Trusts, reconfigure the NHS [8], and accelerate innovation [9] have incentivised hospital mergers. Likewise, the Affordable Care Act in the United States has unleashed “a merger frenzy, with hospitals scrambling to shore up their market positions, improve operational efficiency, and create organizations capable of managing population health” [10]. Therefore, it is likely that in the coming years the trend for hospital mergers and integration will continue on both sides of the Atlantic.

Theoretically, integration through merger can be successful because it is associated with beneficial synergistic impacts, reduced duplication of services, economies of scale and scope (especially management costs), and increased market power [11]. In practice, however, benefits of mergers are “often based on managers’ beliefs about the benefits” rather than evidence [12], and in reality many mergers fail [12-15]. An increasing body of literature suggests that organisational culture affects the success of post-merger integration in healthcare [12,14-20]. For example, extensive multicentre studies of mergers in the UK found that cost savings from these mergers were minimal and that perceived differences in organisational culture form “a barrier to bringing organisations together” [12,14]. A recent overview of hospital mergers in Europe and North America argued that “[a]lmost all consolidations fall short, since those in leadership positions lack the necessary understanding and appreciation of the differences in culture, values and goals of the existing facilities” [15]. Research outside healthcare also highlighted the role of cultural compatibility in successful post-merger integration and called for cultural due diligence [21-23]. In particular, KPMG showed on a global scale that 83% of corporate mergers and acquisitions fail to enhance shareholder value, but that they are 26% more likely to be successful if they focus on identifying and resolving cultural issues [24].

Successful post-merger integration in university hospitals and AHCs is even harder to achieve because universities and hospitals have to integrate their academic and clinical enterprises while maintaining their organisational independence. It is such a formidable challenge that some have argued “[t]o date, an example of a vibrant and successful merger of academic health centers remains to be found” [25]. There is growing literature to suggest that organisational culture plays an important role in inter-organisational collaboration and partnership [26-31]. An analysis of a failed merger in the US concluded that “[w]ithout an exhaustive and in-depth review of organizational culture, mores, values, and mission, perhaps [mergers in academic medicine] are, in fact, destined to be folly” [31]. Likewise, an analysis of successful mergers in the US argued that in all merging teaching hospitals the cultures of legacy organisations do not align and that “[t]he challenge is to understand the degree of gap and how best to manage it over the subsequent process” [20].

Although the role of organisational culture in post-merger integration and inter-organisational collaboration is widely recognised, little empirical evidence exists to help academic and clinical leaders identify differences in culture and resolve cultural issues early in post-merger integration. In this article, we report our empirical findings from a study into organisational culture at one NHS trust during its post-merger integration with another NHS trust and strategic partnership with a university. Given that our analysis is based on the Competing Values Framework, which is connected to a large body of theoretical and empirical literature, our findings will add to an evidence base around this framework, especially in an academic medicine setting, and will allow formulation of hypotheses for future research. Many of our findings will be relevant to academic and clinical leaders in other university hospitals and AHCs contemplating an assessment of organisational culture as a means of assisting successful post-merger integration and academic-clinical collaboration. Our findings will also be relevant to national policy-makers seeking to reconfigure health services and accelerate innovation.

## Methods

### Research setting

This study was conducted at the former Nuffield Orthopaedic Centre NHS Trust (NOC) during the first three months of its post-merger integration with the former Oxford Radcliffe Hospitals NHS Trust (ORH). These two NHS trusts combined at the same time as they undertook a strategic partnership with the University of Oxford, thereby creating the Oxford University Hospitals NHS Trust (OUH).

The NOC was a £79 million turnover single-hospital organisation [32] providing orthopaedic and rheumatologic services on one site near to the Churchill Hospital. The ORH was a £636 million turnover multi-hospital organisation [33] providing a wide range of general and specialist services across three sites: the John Radcliffe Hospital and the Churchill Hospital in Oxford (less than a mile away from each other), and the Horton General Hospital in Banbury (twenty miles away from Oxford). As a result of merger on 1st November, 2011, the NOC joined the OUH as one of its seven clinical services divisions – the Musculoskeletal and Rehabilitation Division – while retaining its name as a hospital. The arrangement is captured well in the new NHS trust’s slogan, “Four Hospitals, One Trust, One Vision”.

The merger integration was envisaged to “make a step change in quality, cost-effectiveness and the academic-clinical integration” as well as “help to ensure the organisations’ long-term financial stability and enhance the ability to achieve Foundation Trust status within three years in line with Government requirements” [34]. Improvements in the quality of care were planned to be achieved through the redesign of care pathways that crossed organisational boundaries. Improvements in cost-effectiveness were expected to come over time from reductions in duplicate activities. In particular, these improvements would be in corporate services in the short term, and from the optimised use of theatres and wards in the medium term [34]. In the long term, improvements would come from an increased number of tertiary referrals, international patients, clinical trials and research opportunities encouraged by the joint NHS/University brand [34]. Importantly, the merger did not seek to make changes to the configuration of clinical services in either of the legacy NHS trusts at the point of merger. It was agreed that any subsequent changes to clinical services and the patient groups to be treated on respective sites would be considered on their own merit, taking into consideration patient interest, after the merger had taken place.

Although in the run up to the merger the NOC implemented a successful turn-around programme to make substantial efficiency savings and generate additional income, it would struggle to achieve Foundation Trust status and maintain its financial viability in the long run because, unlike the ORH, the NOC did not have the breadth of the services that were sufficiently funded. The NOC had predominantly specialist services, which were not fully paid for under the Payment by Results (PBR) reimbursement system. Moreover, taking into account the diminishing cash envelope from the commissioners, the NOC had to deal with the stranded costs of the new buildings and infrastructure funded through the Private Finance Initiative (PFI). In such circumstances, the NOC’s Board of Directors felt that the best way to secure the NOC’s long-term interests was to enter into a voluntary merger with an organisation that shared its priorities and, thereby, to be an active member in the merger process rather than face the prospect of losing specialist services and be deemed non-viable in the future.

Historically, clinical collaboration between the NOC and the ORH was limited, but they both had a strong tradition of successful academic-clinical collaboration with the University of Oxford. The NOC had built its reputation as the UK’s leading specialist provider of musculoskeletal clinical care [35] on its own and throughout its history remained fiercely independent, whereas the constituent hospitals of the ORH worked together with many other organisations in the local health economy. Most strikingly, between 1948 and 1974, all Oxford hospitals except the NOC formed a Teaching Hospital Group known as the United Oxford Hospitals [36].

Nevertheless, both the NOC and the ORH had a long tradition of successful academic-clinical collaboration with the University of Oxford, and had a common benefactor. In the 1930s, Lord Nuffield’s benefaction helped modernise the NOC and establish university clinical departments in the hospitals belonging to the ORH and the NOC [36,37]. At the point of merger, ten university clinical departments were co-located and embedded within the ORH and one within the NOC; and University-employed clinical academics made a significant contribution to the provision of high-quality health services by both NHS trusts. Successful academic-clinical collaboration between both NHS trusts and the University led to the establishment of the National Institute for Health Research (NIHR) Oxford Biomedical Research Centre (BRC) at the ORH in 2007 [38] and the NIHR Oxford Musculoskeletal Biomedical Research Unit (BRU) at the NOC in 2008 [35]. Since 2009, Oxford has been the UK’s leading centre for academic research in clinical medicine as measured by research income [39].

The Joint Working Agreement between the University and the merged NHS trusts also came into being on 1st November, 2011, effectively providing a formal institutional framework for one of Europe’s largest and most research-intensive AHCs:

- The Agreement institutionalised a strategic partnership between the academic and clinical partners with a joint tripartite mission of patient care, education, and research.
- The partners established joint governance structures, including a Strategic Partnership Board and a Joint Executive Group with four specialist committees.
- The partners entered into a Trade Mark Licence, which paved the way for the joint NHS/University brand identity. The license governs the use of the University mark in the NHS trust name in relation to the supply of health services.
- In the run-up to the Agreement, the clinical (NHS) partners introduced a clinically-led management structure that aligned with university clinical departments and thus enhanced academic-clinical collaboration.
- The partners pooled their resources and co-located their clinical trials and research governance teams to create a Joint Research Office.

### Research design

The University of Oxford Clinical Trials and Research Governance Team reviewed the study, and it deemed that no further ethics committee clearance was necessary. An anonymous online survey was conducted in October-November 2011 among 72 clinician-scientists at the NOC, who constituted the entire population of clinician-scientists jointly employed by the NOC NHS Trust (as it was then) and the University. We anticipated that one of the drawbacks of the survey among clinician-scientists might be a low response rate. In order to maximise it, we employed a number of methods based on a systematic review of evidence: a short questionnaire, a personalised mailout, a clinician-scientist contact, and subsequent reminders [40].

The focus of the study on clinician-scientists was chosen to increase its validity, reliability, and cost-effectiveness, as well as to enable comparison with previous research. Since the clinician-scientists in our study population worked in both organisations, they were in the best position to assess the pre-merger cultures of their NHS Trust and University, as well as the preferred future NHS Trust/University culture. The size of the population of clinician-scientists made it possible to maximise the response rate and minimise the sampling error by surveying the entire study population in a fast and cost-effective manner. Given that different groups of staff may perceive organisational culture differently, the focus on clinician-scientists also helped ensure the homogeneity of the study population and the reliability of the quantitative and qualitative data collected. The focus on clinician-scientists at the NOC also enabled a comparison with previous research at the ORH, which focussed on clinician-scientists.

**Figure 1:**
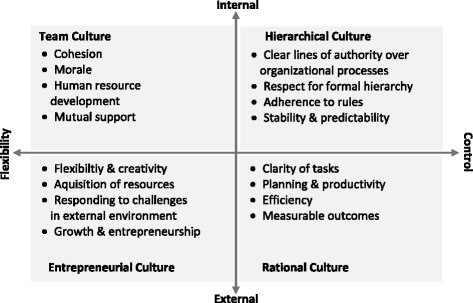
**The Competing Values Framework.** Adapted from: Helfrich CD, Li YF, Mohr DC, Meterko M, Sales AE: Assessing an organizational culture instrument based on the Competing Values Framework: exploratory and confirmatory factor analyses. *Implementation Science* 2007, 2:13.

The survey instrument was adapted from the US Veterans Affairs Administration All Employee Survey and included 14 organisational culture items grouped into four subscales corresponding to the four cultural archetypes of the Competing Values Framework (CVF) [41]. Among the many ways to measure organisational culture in health services research [42,43], the CVF is the method used most frequently [41]. It distinguishes between two dimensions of an organisation’s competing or opposite values/priorities: centralisation and control *versus* decentralisation and flexibility; as well as the internal environment and processes *versus* the external environment and relationships with outside stakeholders. The resulting quadrants of the framework represent four cultural archetypes – entrepreneurial (also known as developmental or adhocracy), team (group or clan), hierarchical, and rational) – which are depicted in Figure 1 together with their major characteristics. Respondents were asked to indicate on a five-point Likert scale ranging from 1 (strongly disagree) to 5 (strongly agree) the extent of their agreement or disagreement with the items concerning both the current, i.e. pre-merger, culture in their NHS Trust and University and the preferred future NHS Trust/University culture, i.e., what the culture across the two organizations should be like in five years in order to more successfully pursue the mission of academic medicine.

As has been argued elsewhere [29], the advantages of the CVF are that it focusses on an organisation’s key cultural characteristics, measures organizational culture in a standardised way, and connects to a large body of theoretical and empirical literature on organisational culture and performance [44-60]. Empirical evidence suggests that patient satisfaction is positively associated with team culture and negatively with hierarchical culture [46]; safety is positively associated with team and entrepreneurial cultures and negatively with hierarchical culture [50]; and physician job satisfaction is positively associated with team culture and negatively with hierarchical culture [52]. Research among non-supervisory employees shows that healthcare facilities belonging to the US Veterans Affairs Administration are characterised by dominant hierarchical culture, strong rational culture, and weaker team and entrepreneurial cultures [41]. At the same time, research among senior managers shows that UK NHS trusts are most frequently characterised by dominant clan (i.e. team) culture, strong rational culture, and weaker hierarchical and developmental (i.e. entrepreneurial) culture. The corollary is that different NHS trusts may have different cultures, and different groups of staff may perceive organisational culture differently.

In order to counter the drawbacks of quantitative methods, the study also employed qualitative methods. Besides the CVF instrument, the survey included three items prompting respondents to provide any additional open-ended comments or thoughts on the major cultural issues for the NOC/ORH merger, and its impact on academic-clinical integration. To preserve the anonymity of responses, the survey included a link to another online form, where respondents could also submit their email address if they were willing to be approached for interview.

Semi-structured interviews were conducted with six self-selected clinician-scientists at the NOC in January 2012 (Additional file 1). Interviews were on average 67 minutes in length, and conducted in the interviewee’s environs. They explored organisational culture through interviewee descriptions of their work and its position within their field, hospital, and academic-clinical collaboration; opinions as to the impetus for merger, its conduct and potential cultural, clinical and academic effects; experiences of academic-clinical collaboration; and opinions of the Joint Working Agreement. The interviews were digitally recorded (4 hours 41 minutes in total), transcribed (167 pages in total), and anonymised.

The interviews and open-ended responses from the survey were then classified and analysed inductively for emerging themes. Researchers were informed by the CVF characteristics (Figure 1), previous research on organisational culture at the ORH [29], and the themes emerging from the earlier qualitative work on “Aligning Excellence”, which sought to identify and extend good practice in medical research and patient care across the NHS trusts and the University [61]. Deductive reasoning was used to link emerging themes with the four cultural archetypes of the CVF.

During the analytical stage of the study, one of the authors (JF), who is the former Chief Executive of the NOC, held a group discussion with other former members of the NOC Executive Team to corroborate and explain the findings from the survey and interviews. Notes of the main discussion points were taken and incorporated in the introduction and discussion sections. Insights from the executives and the perceptions of clinician-scientists are presented separately to complement each other.

While analysing and interpreting the mixed-methods quantitative and qualitative findings from the survey and interviews, we treated both types of findings as complementary rather than competitive. As argued by Moffatt *et al.* this allows researchers to exploit the strengths of both quantitative and qualitative methods as well as to counter the limitations of each [62]. The advantage of reporting both qualitative and quantitative data together without making assumptions about the “correct” data is that this approach contributes to “increasing the likelihood of arriving at a more thoroughly researched and better understood set of results” [62].

## Results

**Figure 2:**
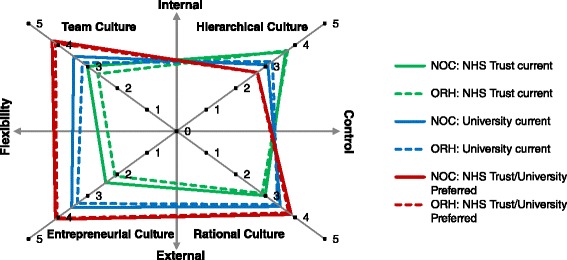
**Organisational culture profiles of the current (pre-merger) cultures at the two merging NHS Trusts, University clinical departments, and the preferred future NHS Trust/University culture, according to 2010 (ORH) and 2011 (NOC) organisational culture surveys.** The preferred future NHS Trust/University culture refers to the culture that should be developed across the clinical and academic enterprises in the next five years to more successfully pursue the shared mission of academic medicine.

A total of 38 completed questionnaires (response rate = 53%) were received. Scores for each item were calculated by averaging individual responses, and scores for each culture subscale by averaging questions on the culture subscale (Additional file 2). The reliability of our results, as measured by Cronbach’s α, was highest for the entrepreneurial subscale, moderate for the team subscale, and lowest for the rational and hierarchical subscales (Additional file 2). To demonstrate the current organisational culture of the NOC NHS Trust and the University, as well as the future preferred organisational culture across the two organisations, we plotted the results from the NOC against the results from the ORH on the CVF axes (Figure 2). It is interesting to note the key differences and similarities in the perception of organisational culture between the NOC and the ORH:

- respondents at the NOC perceived that its clinical culture was more team-oriented and entrepreneurial than at the ORH (P < .05; Additional file 2) and as hierarchical and rational as at the ORH (P > .05; Additional file 2);
- respondents at both the NOC and the ORH perceived that the academic enterprise had a less hierarchical and more team-oriented, entrepreneurial, and rational culture than the clinical enterprise;
- respondents at both the NOC and the ORH perceived the current University culture and the future preferred NHS/University culture almost identically.

Because of the small sample size at the NOC, we did not perform a comprehensive statistical analysis, and instead concentrated on the analysis of qualitative data from the survey and interviews. Of the respondents, 24 (33%) elaborated on cultural items from the questionnaire or other issues of particular concern. These helped to shape the avenues of investigation in interview, and opinions expressed were in large part replicated – in greater detail – in interviews. We provide below our analysis of qualitative data with a selection of the most informative respondent and interviewee quotations to illustrate the range and depth of perspectives and to highlight major cultural issues and potential problems.

### Entrepreneurial culture

The NOC was perceived by its members to be more research-active and more entrepreneurial than the ORH. Those interviewed attributed this to the nature of their clinical area and the loyalty of patients with chronic conditions to the institution and its research ventures. Because of its smaller size, the NOC was also perceived to be more flexible than the ORH, but there was a concern that in the merged organisation this would be lost:

- “The [merged] organisation is so complex that it becomes very difficult to change things. I am very concerned that NOC will get dumbed down and clinical enterprise and innovation lost”.
- “It’s just that you have to adapt yourself to be effective in a different organisational culture”.

However, the clinical enterprise at the NOC was perceived as less flexible and entrepreneurial than its academic enterprise, mainly because the NHS in general was thought to be too risk-averse, over-regulated, and focused on finances and immediate clinical impact:

- “It is very difficult to find anyone prepared to be responsible for a change in practice [in the NHS]. Managers get kudos for organisational changes which are often not in anyone’s interest, but difficult to stop. There is often blind adherence to directives. University is a bit more flexible, especially on the HR front, allowing short term employment”.
- “It is critical that the metric of improving patient care be the main one used to drive innovation. It is not possible to complete every single regulatory dictate to the letter and still have time to produce research that improves patient care. …The job of the regulation is not to provide a zero risk environment for patients but to balance it with innovation that may improve care”.

Those interviewed welcomed the Joint Working Agreement because it provides a formal institutional framework – and thereby important support – for current academic-clinical collaboration. Nevertheless, a major issue for respondents was to reconcile different priorities in academic and clinical innovation and service delivery. There was a clear recognition of the different roles and primary emphases of clinical and academic settings and the need to balance these:

- “University is intensely innovation focused and has to continue this to remain competitive. ORH is service delivery orientated and has to continue this to remain viable. What works for one will not always work for the other and there is some complete and unavoidable incompatibility between the goals of both organizations. These need to be identified and coping strategies put in place so that time is not wasted re-identifying the same clash in different formats”.
- “NHS pathway redesign may preclude easy data access to research subjects if increase same day admission, one stop shop, etc. Enabling research access may decrease the efficiencies that can be achieved on operational NHS delivery. Cultures of both organisations need to understand importance of symbiotic working”.

### Team culture

The NOC was perceived to have a more team-oriented culture than the ORH because it was a relatively small organisation, and staff had a shared vision and were proud of their organisation. A statement by one individual was echoed in all interviews: “The NOC is about excellence and quality in orthopaedics”. This clarity of vision gives insight into the strong identity and loyalty the NOC enjoys and would like to preserve:

- “Staff here [at the NOC] are hugely proud of their low infection rates, of hygiene, of MRSA, of service delivery and of finances. It has run as a very competent little place”.
- “[A major cultural issue for the NOC/ORH merger is] maintaining NOC's identity and staff morale as a special place for excellent patient care, training and academic innovation”.

Although respondents felt that the NOC needed better engagement with the ORH, they were concerned about the NOC being treated unequally and losing its strong team values:

- “[A major cultural issue for the NOC/ORH merger is] ensuring equality across the organisations, the NOC has traditionally been quite introverted and has had no real need to engage with the acute trust [ORH]. Both organisations will need to engage with the issues faced by both – musculoskeletal services are very different on the two sites, and have very different needs”.
- “Ensure that the friendly, approachable attitudes and supportive culture is not lost”.

While respondents in different clinical teams had different – sometimes diametrically opposite – experiences of working with managers, the merger put additional strain on clinician/manager relationships:

- “My clinical managers within the NOC are excellent, very caring and supportive, setting appropriate goals and emphasizing excellence. The [name omitted] department at the NOC has recently been in a state of upheaval, with difficulty getting people to commit to helping sort out problems or even return emails… This may be because of the merger coming up. In previous years, [this department] at the NOC has been fine”.
- “I am very demoralised by the local NHS management… as a clinician I am clearly on my own with very little support. I cannot emphasise enough how negative my responses could be”.

Notwithstanding such marked variations in clinician/manager relationships, respondents generally thought it was easier to engage with managers at the NOC, and they would have preferred a more caring and supportive attitude from managers, better communication, and more teamwork instead of reporting relationships:

- “ORH management is in my experience difficult to engage with as they already have too much on their plates. The NOC management has been more focussed and flexible".
- “Managers and clinicians need to be on the same side; ‘we’, not ‘you’ should be heard much more. Managers and clinicians are together responsible, and neither should hold the other ‘to account’”.

Because the relationship with the University of Oxford is one of partnership rather than merger, concerns with respective team cultures did not arise in the same way as between the NOC and the ORH. Parties to academic-clinical collaborations built their relationships on the basis of – but at the same time independent from – their institution’s team values. That is, collaborations were seen to be between individuals, between researchers or groups, and not between the institutions by which they were employed. Further, many collaborations – especially those undertaken through the NIHR Oxford Musculoskeletal Biomedical Research Unit (BRU) – had been successful long before the Joint Working Agreement, and the process of merger generated uncertainty around research funding:

- “I feel that those in the different organisations who need to communicate and/or collaborate have been doing so over the years anyway”.
- “Uncertainty over management/lines of responsibility for current clinical activities that contribute to research funding uncertainty”.

The majority of respondents were positive about the impact of the merger on academic-clinical collaboration and teamwork. While university-employed clinical academics hoped that it would substantially improve the perception of clinical academics as “valued members of the team”, NHS-employed clinicians hoped to feel more “pulled into the University”. Yet, a minority of NHS clinicians felt disenfranchised and isolated and were concerned that they would lose out to university clinical academics in terms of prestige and opportunities:

- “I have found the University to be rather isolationist, unwilling to include NHS staff in research projects etc., but always wanting the NHS to produce the data for projects and grant applications”.
- “I am very concerned that as a busy clinician with only a small research/teaching component I will be treated on a second tier compared to an academic appointment”.

Respondents repeatedly emphasised that both the NHS and the University urgently needed to pay more attention to staff support and development:

- “In both NHS and University there is a low level of attention to staff development, particularly of the non-clinical staff. Despite this there is a relatively high level of loyalty to the NOC, this loyalty and commitment is at risk if the non-caring attitude of management continues”.
- “The University could learn a thing or two about the value of people and not always doing things just to innovate and ‘be first’”.

### Hierarchical

Those interviewed believed that the NOC had a lesser hierarchical culture compared with the ORH because of the NOC’s smaller size and greater team values. A major concern for post-merger integration was the danger of being “swallowed up” in a larger bureaucracy. Interviewees noted, however, that the ORH could have been perceived as much more bureaucratic simply because it was bigger and unfamiliar. At best, respondents hoped it was simply a matter of learning a new system, but this was tinged with a sense of loss; they had given up something that worked well for them:

- “NOC is a small ‘family’ – with relatively little bureaucracy and a friendly approach to performing day to day tasks, i.e. chat in the corridor, actions taken. This may be at risk if staff turnover/rotation high”.
- “There is concern that the familial environment of the NOC will be eroded by the merger. ORH is perceived to be a large inflexible juggernaut, concerned only with its own priorities”.

Interviewees recognised that the best safeguards against the loss of the familial environment of the NOC were to be found in a devolved organisational structure. The NOC maintained its name as a hospital, and its clinical distinctiveness as one of seven devolved divisions within the new organisation. These were repeatedly emphasised by those interviewed:

- “Of things that were absolutely non-negotiable… one was the name”.
- “As a division, we are not the smallest of the divisions”.

Interviewees maintained that the NOC and the University had distinct but related missions, and systems of governance that reflected these differences. They did not deem these differences as a hindrance and accepted the need to learn how to operate effectively within the two systems. Most believed that the NOC, and the NHS in general, were much more hierarchical than the University, yet some commented that the politics in the University could be as counterproductive as the hierarchy in the NHS:

- “The NHS is target driven from central Government. The talents and time of many able individuals is sometimes wasted in meeting these goals, particular where the benefit to patients is in doubt. Conversely, the University fosters innovation and allows individuals more freedom to excel in their areas of interest”.
- “The politics in the University appear even more divisive than they are in the NHS…this is very damaging for innovation as well as staff development”.

Integration of the NHS trust and University clinical trials and research governance teams is one aim of the strategic partnership. Those interviewed welcomed the idea with caution, still noting that change, however promising an opportunity to reduce bureaucracy, was still difficult and disruptive at the outset. For instance, research application processing times had initially increased:

- “I don’t necessarily know that things were done hugely differently. We all do the same key… GCP [Good Clinical Practice] training and we all deal with the same ethics committees, and so it’s more that it has just ground to a halt in terms of how long it takes to get anything through”.

In the same way that there are adjustments to be made to enable joint and more efficient research administration, there is recognition of the need to align NIHR research infrastructure:

- “There’s a younger generation who see the business necessity [of partnership and integration]. It was interesting to watch [the NOC’s] BRU and [the ORH’s] BRC renewal. This time the bids were put forward with the BRC and BRU knowing what each other was doing. It’s encouraging because it means that people are losing a little bit of an empire mindset and having a joint business plan instead. You just are not going to be competitive if it looks like you can’t talk to your neighbour”.

### Rational

Respondents felt that it was easier to engage in the planning and implementation of the organisation’s goals and objectives with managers at the NOC because ORH managers already had too many responsibilities. The view was that it is necessary to have a more efficient process for scaling up the NOC’s best practices to the merged NHS trust:

- “…even though the ORH has the largest [name omitted] programme there was no unified inpatient [name omitted] service. Instead of saying to us, great let’s use what you have got to save time and get on with it, there was a painful ongoing deconstruction and now reconstruction of the process”.

There was a general feeling among clinician-scientists that the pursuit of greater savings, inadequate reimbursement for specialist services through the Payment by Results (PBR) system, and the increasing costs of repayments on Private Finance Initiative (PFI) capital projects, put additional strains on the post-merger integration with the ORH:

- “Staff at the NOC feel that we have been through many painful rounds of austerity measures in recent years in order to balance our books and are now joined with an organisation which has yet to start this process and is heavily in debt. The fear is that further such rounds will come and be applied equally to all divisions, which will disproportionately affect the NOC”.
- “Risk of losing specialist services, unless fundamental funding flaws of PBR under-reimbursement, and PFI (over-costed due to paying interest on part of hidden national debts) are addressed”.

The majority of questionnaire respondents and interviewees were positive about the formal Joint Working Agreement and saw it as a potential basis for improving clinical services, research, and teaching:

- “I think that there will be an integrated strategy with the three strands [clinical, research, and teaching], as opposed to three completely different strategies”.
- “Increased academic input helping clinicians to measure outcomes and improve practice, and increase profile of the NOC”.
- “On both sides [clinical and research] there is also an increasing awareness about the patient centred-ness of it. Patients expect to have a strong say on what they want for the future”.

A minority of respondents and interviewees had a negative or neutral outlook, however. Some were concerned that one party to academic-clinical collaboration would benefit at the expense of the other. Some felt that “nothing will change and the NOC will carry on just as before”, or that adverse financial conditions would undermine the potential benefits of the merger and strategic partnership:

- “I think that the merger could constrain academic freedom, yet, if managed well, could free up the academic side to undertake higher-impact scientific endeavours”.
- “The merger has no definite clinical benefits for the NOC but there are benefits for the University. Clearer demarcation of funding streams to University or NHS work would be an advantage”.
- “Potentially could be greater true integration and cooperation between NOC and ORH and University for service, teaching, training and R&D innovation. Sadly initial responses, in face of massive savings to be made, resulting in cuts to SPA [standard programmed activity] time etc., indicate the reverse will be true, as doctors retreat into silos to defend their positions in the increasingly hostile environment”.

Those clinician-scientists who participated in the study believed that in the current adverse financial situation, strong and fair leadership was required both locally and nationally, and expressed hopes that clinical leadership would be promoted. They commented positively on the changes in the ORH Executive Team that preceded and enabled the merger:

- “Under the old [ORH] exec team as was five years ago, I don’t believe we would have pursued a merger with them. That changed”.
- “Strong fair leadership will be critical at this difficult time of change locally and nationally for the NHS and academic medicine”.
- “Many clinicians are hoping that the management structures of the NHS are rebalanced towards enabling clinical leadership”.

## Discussion

### Main findings and implications

As the number of mergers involving university hospitals and AHCs is set to grow, academic and clinical leaders are looking for new approaches to ensure success of post-merger integration and academic-clinical collaboration. The main contribution of this article is of an empirical nature. We used a mixed-methods organisational culture approach to examine the perceptions of the pre-merger and the preferred future culture at one of two NHS trusts during their post-merger integration and strategic partnership with the University of Oxford. We identified key differences and similarities in the perceptions of pre-merger culture across the two merging NHS trusts and the University, as well as a number of cultural issues that have important implications for the success of post-merger integration and for strategic partnership with the University.

Qualitative responses indicated that the pre-merger culture of the clinical enterprise at the NOC differed from that at the ORH in a number of ways. Respondents perceived the NOC to be more team-oriented and entrepreneurial, as well as less hierarchical. Qualitative responses regarding rational culture were inconclusive, as respondents did not provide many comments on the differences and similarities in rational culture, and instead concentrated on the general contextual factors related to rational culture. Respondents at the NOC were particularly concerned about losing their identity and familial environment following the merger, and also feared that in the merged organisation enterprise and innovation would be lost to complexity and bureaucracy. According to the NOC Executive Team, the size and scale of the NOC made it possible to develop a culture of informal contact and accessibility. Managers and clinicians were able simply to call in to the office of the Chief Executive and other members of the Executive Team, have a conversation in the corridor, or at the coffee stand. Face-to-face contact made clinicians feel that they were able to get answers, that communication was easier, and that they were able to influence and be heard in a way that is much more difficult to achieve in a bigger organisation. At the same time, qualitative insights from our previous research at the ORH suggest that parties in both NHS trusts share common challenges such as paying more attention to staff development, working in partnership with managers, and overcoming the negative effects of current adverse financial conditions.

Although the NOC sample is not large enough to draw firm comparisons with the ORH, it is important to note that the qualitative results from the NOC support the quantitative finding that the NOC is more team-oriented and entrepreneurial, may support the quantitative finding that both NHS trusts have the same level of rational culture, and do not support the quantitative finding that the ORH is more hierarchical than the NOC. We hypothesise that this is either because the small sample size did not allow reliable quantitative measurements, the NOC personnel misapprehended the relative hierarchicality of the NOC and ORH, or there are problems with the validity of the CVF instrument. Alternatively, the quantitative results may reflect the fact that both NHS trusts shared the same systems of governance and standard operating procedures affecting more deeply-rooted perceptions of organisational culture, whereas the qualitative results may reflect more transitory perceptions of the work environment.

Pre-merger cultures of the clinical enterprise at both the NOC and the ORH are primarily distinct from the academic enterprise, suggesting that clinician-scientists work across two different cultures and that there is a formidable challenge in aligning these cultures to manage this cultural diversity. Notwithstanding the limitations of the small NOC sample for drawing comparisons with the large ORH sample, it is interesting to note that the quantitative results from both NHS trusts support the qualitative finding that the culture of the clinical enterprise is primarily distinct from the culture of the academic enterprise. However, because the relationship between the clinical and academic enterprises is one of partnership rather than merger, there is an acceptance of needing to learn how to operate effectively in these two different cultures. Indeed, as many pointed out, they have long been doing so in their pre-Agreement collaborations. Insights from the NOC Executive Team reveal that since the NHS is a centrally run and funded health system there are indeed people in the NHS who feel that they have to perform certain tasks and duties because of central targets. Therefore, it would be desirable to enhance the culture by moving away from the hierarchical culture towards a more team-based and rational culture, where people would feel engaged and supported, and where entrepreneurial culture could flourish as well. However, the university-type entrepreneurial culture based on individual achievements and governance structures without clear reporting lines and accountability would not be optimal for health service delivery. Our qualitative findings suggest that major issues for respondents are how to reconcile different priorities in academic and clinical innovation and service delivery, how to build inclusive teams, and how to enable “symbiotic working” between the academic and clinical enterprises.

The Joint Working Agreement served as an important ameliorating consideration in reaching merger and holds promise as a common relationship schematic by which to address differences in organisational culture for successful post-merger integration. In so doing, it is important to ensure that despite their smaller size, the academic enterprise at the NOC is as influential as its clinical enterprise, and that the NOC (as was) is as proportionately influential as the former ORH in its relationship with the University. Moreover, it is imperative to develop more efficient processes for sharing and extending best practices between the former NHS trusts, while recognising that there are constraints on the extent to which some best practice can be shared and scaled up. In particular, the NOC Executive Team stressed the importance of getting the right balance and understanding between a more entrepreneurial university culture and the constraints within which the NHS operates. Being a statutory public organisation governed through contracts with healthcare commissioners, any NHS trust has to deliver services that are required by commissioners in accordance with the health needs of the local population. An NHS trust cannot choose to focus on a particular group of patients or a particular condition because of its interest and research potential. Likewise, an NHS trust cannot prioritise the likelihood of innovation over the need to provide good standards of service and to comply with various safety regulations. These constraints make some aspects of entrepreneurial culture difficult to reconcile with the NHS service delivery model, and the high levels of entrepreneurial culture observed in the University may not be attainable in the NHS. Nevertheless, the influence of that culture may serve to encourage what entrepreneurialism is feasible and beneficial in a clinical context.

The merger was viewed as a necessity, but also one with some promise. The majority of respondents detailed a movement from rejection, to resistance, to a gradual willingness to enter into merger. Whilst the long-term goal for the NOC and the ORH to come together was shared by many in the local health economy, including the Strategic Health Authority and commissioners, the NOC Executive Team stressed that the NOC could not have contemplated a merger until the new leadership of the ORH started to change it, and it developed to the point where it became in the interests of both organisations to come together. Also, there was a clear sense of the changing landscape in clinical research and service provision, and of the need to develop a common identity with the University. However, there is still a minority who feel demoralised and disenfranchised. They were particularly concerned with the dangers of receiving very little support from managers, the NOC losing its identity and clinical distinctiveness, and NHS clinicians losing out to university clinical academics in terms of prestige and opportunities. These concerns need to be addressed urgently through effective staff engagement strategies.

We found that changes aimed at strengthening translational research and NHS/university collaboration were disruptive at the outset, but that those who needed to collaborate had been doing so anyway. Respondents particularly stressed the importance and positive impact of the NIHR Oxford Musculoskeletal Biomedical Research Unit (BRU) for translational health research and innovation across the academic and clinical enterprises at the NOC. A similarly positive impact of the NIHR Oxford Biomedical Research Centre (BRC) was found at the ORH [29]. Moreover, the Joint Working Agreement itself evolved from the joint governance arrangements for the NIHR Oxford BRC and BRU. Mergers of university hospitals with existing NHS/university collaborations and proposals for new collaborations should be assessed as to whether they add value to the existing collaborations in the long run, and any such merger should try to minimise the disruption at the outset.

History shapes perceptions of organisational culture and successful post-merger integration. The history of separateness and lack of collaboration between the NOC and the ORH has created memories and stereotypes that negatively affect the staff’s attitudes towards integration and collaboration. According to the NOC Executive Team, the NOC was historically perceived by many in the local health economy as not just separate but isolated; an ivory tower, and not a team player. In turn, the ORH was historically perceived by many NOC members as the “big beast on the hill”: not well-managed and consuming all the attention and resources, as opposed to the “small and beautiful” NOC. At the same time, the history of the NOC’s success while being a separate organisation has helped the staff develop a strong shared vision, identity, and loyalty to their organisation that positively affect staff engagement. Likewise, the history of successful academic-clinical collaboration with the University of Oxford, as exemplified by the NIHR BRU, helped undertake strategic partnership with the University. The latter served as an important ameliorating consideration in reaching the merger. Preserving identities of the merging organisations within a devolved organisational structure is likely to have a positive impact on staff engagement.

Finally, the national policy context played a major role in setting the agenda for the merger as well as in influencing the post-merger integration and strategic partnership with the University. The government policy of designating Academic Health Science Centres every five years provided incentives for the two NHS trusts to consider merger and to formalise their strategic partnership with the University through the Joint Working Agreement. Yet, the major driver for the merger was the government requirement for all NHS trusts to achieve Foundation Trust status. Given that the government repeatedly changed application deadlines and rules for Foundation Trust status, it created added uncertainty and complexity. What is more, the current adverse financial conditions and the unintended consequences of government health care reforms threaten to send doctors and academics retreating back into their silos. Strong fair leadership will be required both nationally and locally for the success of mergers and post-merger integration in university hospitals and academic health centres.

### Strengths and limitations

The main strength of this study is that it uses a systematic mixed-methods assessment of organisational culture as a means of assisting successful post-merger integration and academic-clinical collaboration in an AHC. This study provides empirical evidence to help academic and clinical leaders in a given AHC identify differences and similarities in culture across the academic and clinical enterprises and resolve cultural issues early in post-merger integration and strategic partnership with a university. In order to produce more complete knowledge, this study uses a mixed-methods approach that exploits the strengths of both quantitative and qualitative methods as well as countering the limitations of each [62]. The survey achieved a 53% response, which is higher than in our previous study at the ORH [29], and relatively good for surveys involving clinicians. A seminal study of mail surveys published in medical journals found that surveys of physicians had a mean response rate of 54% compared to 68% mean response rate among non-physicians [63]. Taking into consideration that a survey's response rate may indicate the extent of non-respondent bias, any response rate below 100% can be regarded as a survey’s limitation. Therefore, the richness and diversity of the qualitative data counters the limitations of the quantitative survey and provides a degree of validity that cannot be achieved by quantitative methods alone. Given the empirical and methodological strengths of this study, its results can be used to formulate hypotheses for future research and to improve practice. In particular, academic and clinical leaders in other AHCs contemplating merger will benefit from an increased evidence base that the cultures of their legacy organisations may differ, that the CVF instrument may have limitations in AHC settings, and that a mixed-methods approach may enhance the validity of an assessment of organisational culture in an AHC.

This study has several limitations. It is a single-site study analysing the perceptions of organisational culture and post-merger integration in an academic health centre from the perspective of one merging NHS trust, rather than from both. It focuses on one staff group, i.e. clinician-scientists, rather than all staff groups. Surveying and interviewing all staff groups might have yielded different results. Moreover, the CVF instrument did not capture well the historical issues that the former NOC Executive Team deemed to be important for the success of the post-merger integration. As noted elsewhere, the CVF instrument was not specifically designed for academic medicine [29], and there are concerns about the validity of the CVF instrument in non-academic settings as well [41]. The disagreement between the qualitative and quantitative findings regarding hierarchical culture may indicate problems with the validity of the hierarchical subscale. Respondents did not provide many comments on the differences and similarities in rational culture, and instead concentrated on the general contextual factors related to rational culture. These limitations provide further evidence around the validity of the CVF instrument in AHC settings and may support concerns raised by Helfrich *et al.* about the validity of the instrument when applied to non-managers [41]. Therefore, caution should be exercised in generalising the results of this study and in using the CVF instrument in other AHC settings without prior validation.

## Conclusions

The results of this study indicate that the cultures of two legacy NHS trusts differed, and that the cultures of the clinical enterprise at both legacy NHS trusts were primarily distinct from the academic enterprise. There are challenges in preserving a more desirable culture at one of the legacy NHS trusts; enhancing cultures in both legacy NHS trusts during their post-merger integration, and in aligning academic and clinical cultures following strategic partnership with a university. The seeds of success may be found in current best practice, good will, and the fact that respondents at both legacy trusts aspired towards a near-identical ideal of the future preferred culture. Strong, fair leadership would be required both nationally and locally for the success of mergers and post-merger integration in university hospitals and academic health centres. Our findings have important implications for the integration of health care providers and the promotion of NHS/University partnerships that deserve further research. It might examine staff engagement strategies and cultural interventions to manage cultural diversity and expectations. Further research might also evaluate how such strategies and interventions impact on the success of post-merger integration and academic-clinical collaboration.

## Additional files

Additional file 1:
**Interviews and data collected.**
Additional file 2:
**Survey results.**
