# Supplementary material for: Organisational culture and post-merger integration in an academic health centre: a mixed-methods study
Source: BMC Health Serv Res. 2015 Jan 22;15:25. doi: 10.1186/s12913-014-0673-3 (PMC4308851; doi:10.1186/s12913-014-0673-3)
Supplement: Additional file 1: — Interviews and data collected. [file 12913_2014_673_MOESM1_ESM.pdf]

Additional file 1 Interviews and data collected

No.	Respondent role	Interview date	Interview time	Transcribed pages
1	Musculoskeletal science	05-01-2012	82 min	34
2	Surgery	06-01-2012	73 min	30
3	Rehabilitation	09-01-2012	76 min	32
4	Research management and training	12-01-2012	48 min	20
5	Physiotherapy	13-01-2012	68 min	28
6	Surgery	13-01-2012	54 min	23
Total			*401 min*	*167*
