# Supplementary material for: Organisational culture and post-merger integration in an academic health centre: a mixed-methods study
Source: BMC Health Serv Res. 2015 Jan 22;15:25. doi: 10.1186/s12913-014-0673-3 (PMC4308851; doi:10.1186/s12913-014-0673-3)
Supplement: Additional file 2: — Survey results. [file 12913_2014_673_MOESM2_ESM.pdf]

Additional file 2 Means for individual items grouped by culture subscale, Cronbach's α coefficients for culture subscales, and Mann-Whitney U test results, according to the 2010 ORH (n=170) and 2011 NOC (n=38) organisational culture surveys*

Culture subscale†	NHS Trust pre-merger		University pre-merger		NHS Trust/Univ. preferred	
	NOC	ORH	NOC	ORH	NOC	ORH
**Entrepreneurial**	**2.39 α=.83**	**2.08 α=.72**	**3.54 α=.84**	**3.35 α=.73**	**4.09 α=.90**	**4.04 α=.90**
1. My organization is a very dynamic and entrepreneurial place. People are willing to stick their necks out and take risks.	2.34	1.99	3.32	3.18	4.16	4.10
4. Managers in my organization are risk-takers. They encourage employees to take risks and be innovative.	1.92	1.76	3.05	2.86	3.84	3.88
8. The glue that holds my organization together is commitment to innovation and development. There is an emphasis on being first.	2.50	2.08	3.82	3.79	4.16	4.06
12. My organization emphasizes growth and acquiring new resources. Readiness to meet new challenges is important.	2.79	2.50	3.95	3.58	4.18	4.10
Mann-Whitney U test	*p=.039*		*p=.154*		*p=.587*	
**Hierarchical**	**3.68 α=.45**	**3.75 α=.52**	**3.08 α=.70**	**3.24 α=.50**	**2.73 α=.69**	**2.72 α=.53**
2. My organization is a very formalized and structured place. Bureaucratic procedures generally govern what people do.	4.16	4.20	3.18	3.45	2.47	2.43
5. Managers in my organization are rule-enforcers. They expect employees to follow established rules, policies, and procedures.	4.32	4.24	3.34	3.57	3.03	3.04
9. The glue that holds my organization together is formal rules and policies. People feel that following the rules is important.	3.47	3.73	3.03	3.10	2.82	2.82
13. My organization emphasizes permanence and stability. Keeping things the same is important.	2.87	2.82	2.76	2.82	2.58	2.61
Mann-Whitney U test	*p=.393*		*p=.059*		*p=.764*	
**Team**	**3.01 α=.68**	**2.66 α=.53**	**3.47 α=.73**	**3.18 α=.54**	**4.20 α=0.81**	**4.09 α=.78**
3. Managers in my organization are warm and caring. They seek to develop employees' full potential and act as their mentors or guides.	2.37	2.25	3.11	3.01	4.13	4.19
7. The glue that holds my organization together is loyalty and tradition. Commitment to this organization runs high.	3.82	3.42	3.84	3.67	4.29	4.02
11. My organization emphasizes human resources. High cohesion and morale in the organization are important.	2.84	2.31	3.47	2.86	4.18	4.05
Mann-Whitney U test	*p=.022*		*p=.055*		*p=.287*	
**Rational**	**2.99 α=.68**	**2.89 α=.55**	**3.50 α=.36**	**3.41 α=.58**	**3.87 α=.66**	**3.79 α=.67**
6. Managers in my organization are coordinators and coaches. They help employees meet the organization's goals and objectives.	2.53	2.53	3.18	3.05	3.97	3.95
10. The glue that holds my organization together is the emphasis on tasks and goal accomplishment. A production orientation is commonly shared.	3.11	3.11	3.50	3.25	3.79	3.59
14. My organization emphasizes competitive actions and achievement. Measurable goals are important.	3.32	3.03	3.82	3.92	3.84	3.83
Mann-Whitney U test	*p=.462*		*p=.485*		*p=.334*	

* Respondents rated agreement on a five-point Likert scale where 1 = strongly disagree and 5 = strongly agree.

† The item numbers reflect the original order in the survey questionnaire. Bolded statistics are overall aggregates for the corresponding culture subscales. Italicised statistics for the difference between the corresponding NOC and ORH culture subscales are significant at P<.05.
